# Mo_2_TiC_2_/WSe_2_ nanoarchitectures: *in situ* grown nanoflowers for efficient hydrogen electrocatalysis

**DOI:** 10.1039/d5na01182e

**Published:** 2026-02-11

**Authors:** Antonia Kagkoura, Sergii A. Sergiienko, Anastasios Papavasileiou, Jan Luxa, Zhongquan Liao, Zdeněk Sofer

**Affiliations:** a Department of Inorganic Chemistry, University of Chemistry and Technology Prague Technická 5, 166 28 Prague 6 Czech Republic kagkourn@vscht.cz soferz@vscht.cz; b Centre for Advanced Materials Application, Slovak Academy of Sciences Dúbravská cesta 5807/9 84511 Bratislava Slovakia; c Department of Microelectronic Materials and Nanoanalysis, Fraunhofer Institute for Ceramic Technologies and Systems IKTS Maria-Reiche-Str. 2 01109 Dresden Germany

## Abstract

The combination of 2D materials provides a powerful strategy to enhance electrocatalytic hydrogen evolution. Efficient hydrogen electrocatalysis is achieved by interfacing conductive Mo_2_TiC_2_ MXene with catalytic WSe_2_ nanoflowers *via* a one-step hydrothermal route. The hybrid exhibits low overpotential, fast charge transfer, and long-term stability, outperforming pristine components and establishing Mo_2_TiC_2_/WSe_2_ as a promising hydrogen evolution reaction platform.

## Abstract 2

A straightforward solvothermal route yields a Mo_2_TiC_2_/WSe_2_ hybrid with strong interfacial coupling, delivering enhanced HER activity and excellent long-term stability.

## Graphical abstract

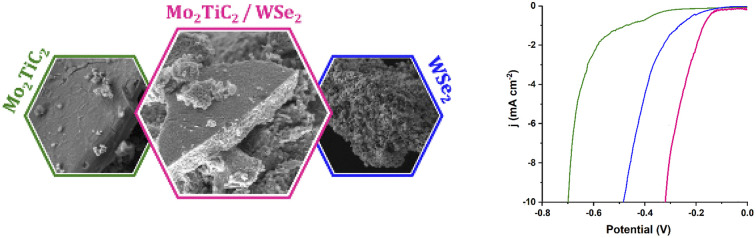

## Introduction

Electrocatalytic water splitting is a key process for sustainable hydrogen production and has attracted significant attention as part of clean energy strategies.^1,2^ The cathodic hydrogen evolution reaction (HER) is particularly important, as platinum remains the benchmark catalyst but is limited by cost and scarcity, motivating the development of earth-abundant alternatives.^3,4^ Two-dimensional materials such as MXenes and transition-metal dichalcogenides (TMDs) have emerged as promising candidates.^5–10^

Among MXenes, Mo_2_TiC_2_ has started to attract attention as the combination of molybdenum and titanium provides high conductivity and a more favorable interaction with hydrogen. In addition, the carbide backbone plays an active role by modulating the electronic structure and providing redox-active sites, further facilitating the hydrogen evolution reaction.^11,12^ Although Ti-based MAX phase MXenes have been widely investigated for electrocatalysis, including HER,^13–15^ MXenes derived from Mo-based MAX phases are still relatively unexplored.^16–18^ The Mo_2_TiC_2_T_*x*_ MXene deriving from the newly synthesized Mo_2_TiAlC_2_ MAX phase offers a unique layered conductive structure with a large surface area, highly desirable for electrocatalysis. The incorporation of Mo not only enhances hydrogen adsorption properties, bringing the binding energy closer to the optimal range for HER, but also provides redox-active sites (Mo^6+^/Mo^4+^) that can serve as catalytic centers, adsorption and electron transfer.^16,19^ This synergy with Ti improves electronic conductivity and structural robustness, leading to superior catalytic activity and cycling stability.^19^ Moreover, Mo_2_TiC_2_ has demonstrated promising activity towards electrocatalytic HER, outperforming several other MXenes and even approaching noble metal catalysts,^20–22^ while also serving as an excellent platform for nanoparticle immobilization.^17,23^

Within the TMD family, WSe_2_ has shown promising HER activity, often matching or surpassing MoS_2_ when optimized for phase and defect structure.^24–27^ Metallic (1T) WSe_2_ exhibits low charge-transfer resistance compared to its semiconducting (2H) phase, and strategies such as defect engineering or doping can further improve proton adsorption and catalytic kinetics.^26,28,29^ These features make WSe_2_ a strong candidate for building hybrids with conductive MXenes,^18^ where interfacial charge transfer can be further enhanced.

A widely used approach to boost catalytic performance is the *in situ* growth of 2D materials on conductive substrates, which not only provides mechanical stability but also enhances charge transport and reduces interfacial barriers during electrocatalysis. This synergy has been demonstrated in hybrids such as WSe_2_/Ti_3_C_2_Cl_2_ in our previous work,^30^ yet no studies have explored the combination of WSe_2_ with Mo_2_TiC_2_, a newer MXene with potentially stronger interfacial interactions. Mo_2_TiC_2_'s distinct electronic structure and surface chemistry enable intimate *in situ* growth of WSe_2_, enhancing interfacial charge transfer and structural stability—features not observed in the more commonly studied Ti_3_C_2_–TMD systems. Compared to Ti_3_C_2_- or V_2_C-based MXene/TMD hybrids, the Mo_2_TiC_2_/WSe_2_ system offers unique electronic properties, with Mo^4+^/Mo^6+^ redox-active sites^31^ (dominant Mo^4+^ with minor Mo^6+^ surface contribution),^32^ facilitating charge transfer and contributing to superior HER performance.

We report the first solvothermal synthesis of WSe_2_ nanoflowers directly grown on Mo_2_TiC_2_ MXene, forming a hybrid with direct interfacial contact. The material shows low overpotential, a small Tafel slope, and improved electrical conductivity, indicating efficient charge transfer and abundant catalytic sites. Stability tests confirm that the Mo_2_TiC_2_/WSe_2_ heterostructure is a durable and effective electrocatalyst for hydrogen evolution.

## Experimental

### General

All chemicals, reagents, and solvents were purchased from Sigma-Aldrich and used without further purification.

### Preparation of Mo_2_TiC_2_ MXene

The initial powders (Ni, Mo, Ti, Al, C) were sequentially mixed with ethanol to obtain a suitable homogeneous mixture and then dried at 60 °C for 2 hours. Initial composition, of the sample was Ni : Mo : Ti : Al : C = 1 : 2 : 1 : 7 : 2. Next, 4 g of powder mixtures were pressed (10 kN) uniaxially in the shape of discs (diameter of 25 mm, thickness approx. 3 mm). The samples were placed on the top of the Al_2_O_3_ powder layer in an alumina crucible and were also covered with an Al_2_O_3_ powder layer to reduce the evaporation of Al. The powder compacts were sintered to form MAX phase/Ni–Al alloy composite using conventional sintering of initially pressed powders in an Ar atmosphere at 1500 °C and for 4 hours, with a heating/cooling rate of 5 °C per minute, synthesis conditions are described in more detail in the publication.^17^

Etching of Al and Ni to form MXene from the sintered and milled sample was carried out in HF solution at 70 °C for 8 days (2 g of powder was added to 40 mL of 50 wt% HF aqueous solution). The etching was carried out in two stages: the first etching lasted 4 days, after which the powder was washed with distilled water to remove AlF_3_. The sample was then subjected to a second etching step for an additional 4 days. As a next step, the obtained powder was washed with distilled water (at room temperature), filtered using vacuum filtration with a filter paper and dried at room temperature.

For delamination, TBAOH (20 wt% in water) was used for 1 day. Next, the samples were washed in distilled water to dissolve TBAOH. Samples in the form of powders were filtered through filter paper and dried at room temperature for 1 day.

### Preparation of WSe_2_

WSe_2_ was synthesized by dissolving 1 mmol of tungsten hexacarbonyl and 2 mmol of selenium powder in 30 mL of dimethylformamide (DMF). The mixture was then transferred into a 50 mL Teflon-lined stainless-steel autoclave and heated at 200 °C for 13 hours. Once cooled to room temperature, the suspension was subjected to centrifugation at 10 000 rpm, followed by washing cycles with DMF (twice), distilled water (three times), and methanol (three times).

### Preparation of Mo_2_TiC_2_/WSe_2_

Tungsten hexacarbonyl (175.95 mg) and selenium powder (78.97 mg), along with 3 mg of Mo_2_TiC_2_ MXene were dissolved in 30 mL DMF and the resulting suspension was transferred into a 50 mL Teflon-lined stainless-steel autoclave reactor and heated at 200 °C for 13 h. After the autoclave was cooled to room temperature, the resulting suspension was centrifuged at 10 000 rpm with DMF (2 times), distilled water (3 times) and methanol (3 times).

### Microscopy techniques

The morphology of the synthesized materials was examined by scanning electron microscopy (SEM) using a Tescan Lyra 3 dual microscope with a FEG electron source. Elemental composition and distribution were determined by energy-dispersive X-ray spectroscopy (EDS) equipped with an 80 mm^2^ SDD detector and analyzed with the AZtecEnergy software package (Oxford Instruments). For SEM/EDS analyses, the samples were mounted on carbon-conductive adhesive tape. Transmission electron microscopy (TEM) investigations were carried out with a Jeol EFTEM 2200 FS microscope (Jeol, Japan) operating at 200 kV. Micrographs were captured using a SIS MegaView III digital camera (Soft Imaging Systems) and further processed with AnalySIS v.2.0 software. TEM including high-angle annular dark-field (HAADF) imaging and high-resolution TEM (HRTEM) imaging were obtained in a Zeiss LIBRA 200 MC Cs STEM, operating at 200 kV. For TEM sample preparation, dispersions were made in absolute ethanol, the suspension was then put on carbon lacey TEM grids (200 mesh, PLANO Gmbh) by pipette, and dried overnight at room temperature.

### XRD

Powder X-ray diffraction measurements were carried out at room temperature using a Bruker D8 Discoverer diffractometer (Bruker, Germany) equipped with a parafocusing Bragg–Brentano geometry and Cu Kα radiation (*λ* = 0.15418 nm, 40 kV, 40 mA). Patterns were recorded within the 2*θ* range of 5–70° with a step size of 0.019°. The acquired data were processed using the EVA software package.

### XPS

X-Ray photoelectron spectroscopy (XPS) measurements were performed using a Phoibos 100 spectrometer (Specs, Germany) equipped with a monochromatic Al Kα1 source (1486.7 eV). Samples were mounted on Cu conductive tape. High-resolution core-level spectra were acquired with a pass energy of 40 eV and a step size of 0.1 eV. Charge compensation was applied using a flood gun, referencing the C 1s peak to 284.8 eV.

### Raman spectroscopy

Raman spectra were collected using an inVia Raman microscope (Renishaw, UK) operated in backscattering geometry and equipped with a CCD detector. A DPSS laser with 532 nm excitation (50 mW source power, 5 mW applied at the sample) and a 50× objective lens was employed. Calibration was performed using a silicon reference with a characteristic peak at 520 cm^−1^, providing a spectral resolution better than 1 cm^−1^. For sample preparation, dispersions (1 mg mL^−1^ in deionized water) were ultrasonicated for 10 min, drop-cast onto silicon substrates, and dried prior to measurement.

### Electrochemical measurements for hydrogen evolution reaction

The electrochemical characterization by means of linear sweep voltammetry (LSV) was performed using an Autolab PGSTAT 204 (Metrohm, Switzerland). A standard three-compartment electrochemical cell was used equipped with a rotating disc electrode (RDE) with a glassy carbon disk (geometric surface area: 0.196 cm^2^) as a working electrode, graphite rod as a counter-electrode, and Hg/HgSO_4_ (0.5 M K_2_SO_4_) as reference electrode. HER LSV measurements were performed at room temperature in Argon-saturated aqueous 0.5 M H_2_SO_4_ solution. LSV plots were corrected for iR drop by applying a 5–10% iR correction to the measured potentials to account for the ohmic losses, as the correction varied depending on the resistance of the samples. For preparing the catalyst ink, catalytic powder (4.0 mg) was dissolved in a mixture (1 mL) of deionized water, isopropanol, and 5% Nafion (v/v/v = 4 : 1 : 0.02) followed by sonication for 30 min before use. The working electrode was polished with alumina suspension, washed with deionized water, and finally sonicated in double-distilled water before casting 8.5 µL aliquots of the electrocatalytic ink on the electrode's surface. Finally, electrochemical impedance spectroscopy (EIS) measurements were acquired from 10^5^ to 10^−1^ Hz with an AC amplitude of 0.01 V. The EIS measurements were conducted at a potential where significant HER current was recorded, corresponding to −2 mA cm^−2^.

## Results and discussion

**Fig. 1:**
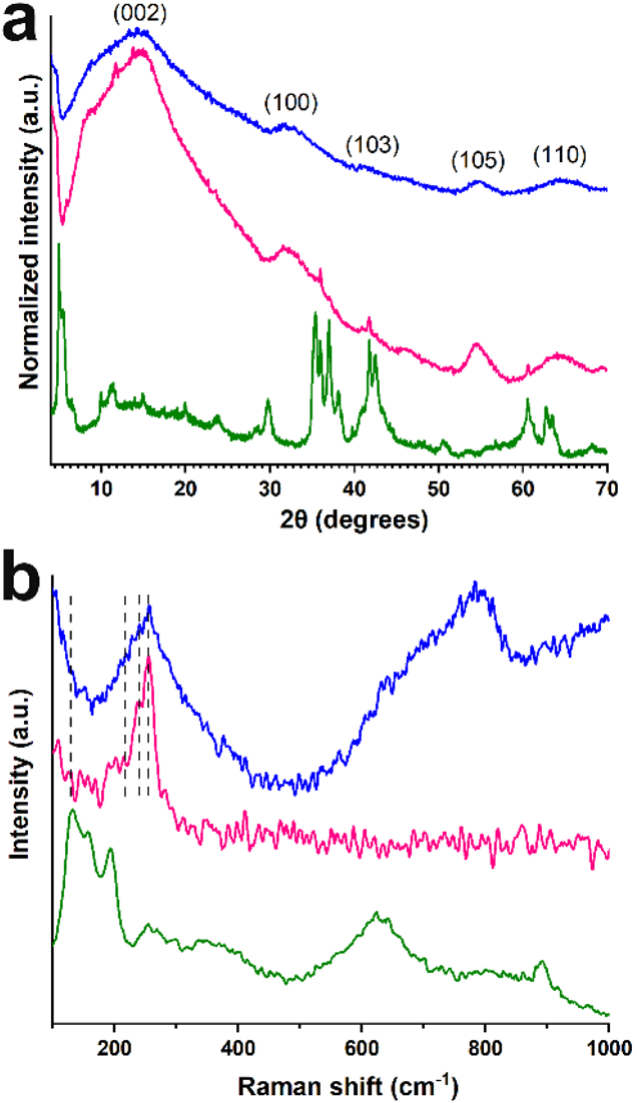
(a) XRD patterns and (b) Raman spectra for Mo_2_TiC_2_/WSe_2_ (pink), WSe_2_ (blue) and Mo_2_Ti_2_C_2_ MXene (green).

The hybrid was prepared *via* a simple solvothermal approach, where WSe_2_ formed on the Mo_2_TiC_2_ MXene, that was used as a platform, through the reaction of tungsten hexacarbonyl with selenium. The Mo_2_TiC_2_/WSe_2_ hybrid was prepared with a WSe_2_-rich composition, following our previous optimization on WSe_2_/Ti_2_C_2_Cl_2_ hybrids, where higher WSe_2_ loading resulted in enhanced HER performance.^30^ The XRD patterns of Mo_2_TiC_2_/WSe_2_ confirm the successful formation of the hybrid structure. Fig. 1a shows the diffraction patterns of the Mo_2_TiC_2_/WSe_2_ along with the pristine materials, highlighting the diffraction planes corresponding to hexagonal WSe_2_ (space group *P*6_3_/*mmc*, JCPDS No. 38-1388). The pristine WSe_2_ exhibits characteristic reflections at the (002), (100), (103), (105), and (110) planes.^33^ In the Mo_2_TiC_2_/WSe_2_ hybrid, the (002) reflection appears less broad compared to the pristine WSe_2_, while additional peaks originating from the MXene overlap in the same region (8.57°, 11.5°). The (100) reflection is downshifted by ∼1°, while the (103) reflection shifts toward higher 2*θ* by ∼1°, indicating subtle lattice distortions associated with interfacial coupling and interlayer strain upon hybrid formation.^30,34^ Additional peaks at 35.9°, 37.0°, 41.67° and 60.7° originating from the MXene phase, are also detected in the hybrid.^33^ XRD patterns of the synthesized Mo_2_TiC_2_ MXene are presented in Fig. S1, with the pristine MAX phase included for comparison.^17^

The Raman spectra of all materials are shown in Fig. 1b. In the Raman spectrum of pure Mo_2_TiC_2_T_*x*_, we can ascribe the band at 158 cm^−1^ to *E*_g_ vibrations from both Mo and Ti atoms in the oxygen-terminated Mo_2_TiC_2_.^33,35^ The *E*_g_ vibration at 254 cm^−1^ can be related to the presence of oxygen. Higher frequencies (360, 420 and 625 cm^−1^) can be attributed to mostly C vibrations in Mo_2_TiC_2_.^33,35^ Intact WSe_2_ shows bands at 131, 212, and 238 cm^−1^, corresponding to *J*_1_, *J*_2_, and *J*_3_, which are characteristic of the metallic 1T octahedral phase of WSe_2_.^30,36^ Additionally, the band at 256 cm^−1^ for WSe_2_ results from the overlapping E^1^_2g_ and A_1g_ modes, typical of the 2H phase.^30,36^ In the hybrid material, bands deriving from both individual components are observed: *J*_1_-*J*_2_ bands are seen deriving from WSe_2_'s 256 cm^−1^ band appears downshifted to 254 cm^−1^ in the hybrid. The latter, along with the observed shift in XRD suggests interfacial coupling between WSe_2_ and MXene layers upon hybridization. This region also overlaps with the C vibrations in the MXene.

**Fig. 2:**
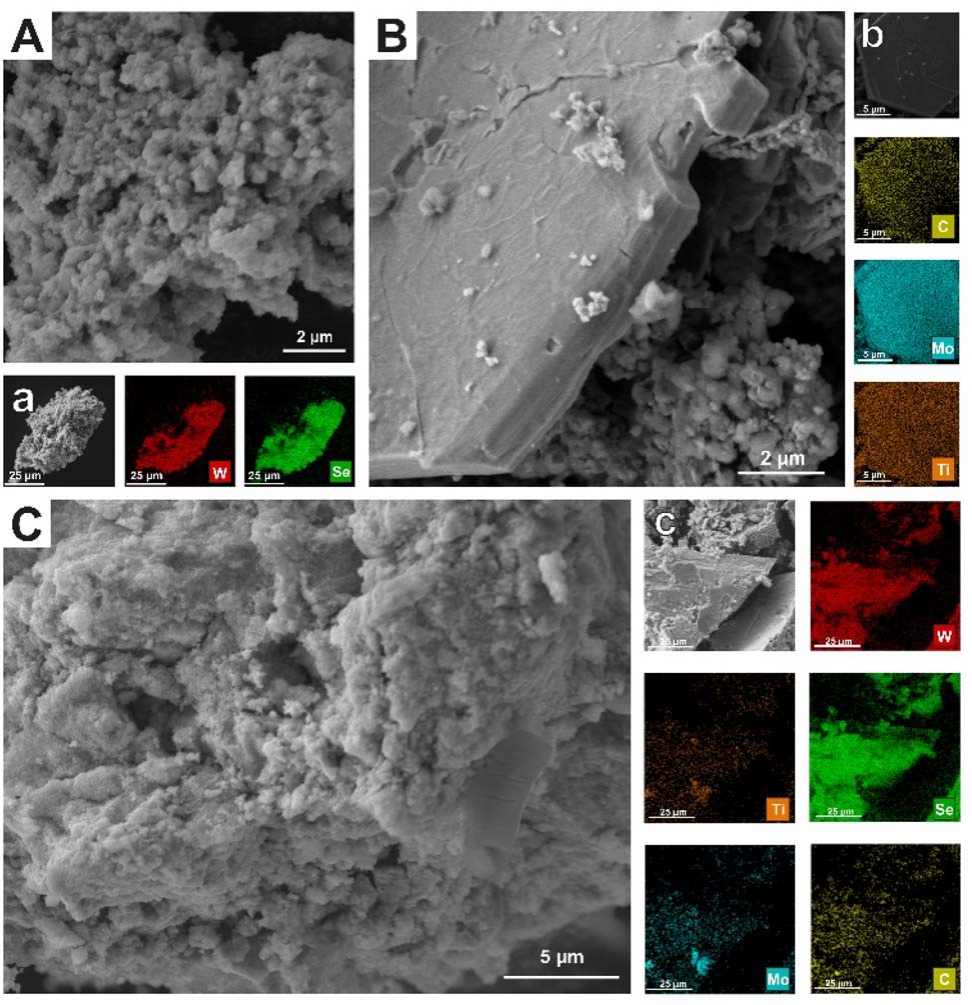
SEM images of (A) WSe_2_ (B) Mo_2_TiC_2_ MXene and (C) Mo_2_TiC_2_/WSe_2_ hybrid with corresponding EDS elemental maps (a–c) showing the distribution of elements.

**Fig. 3:**
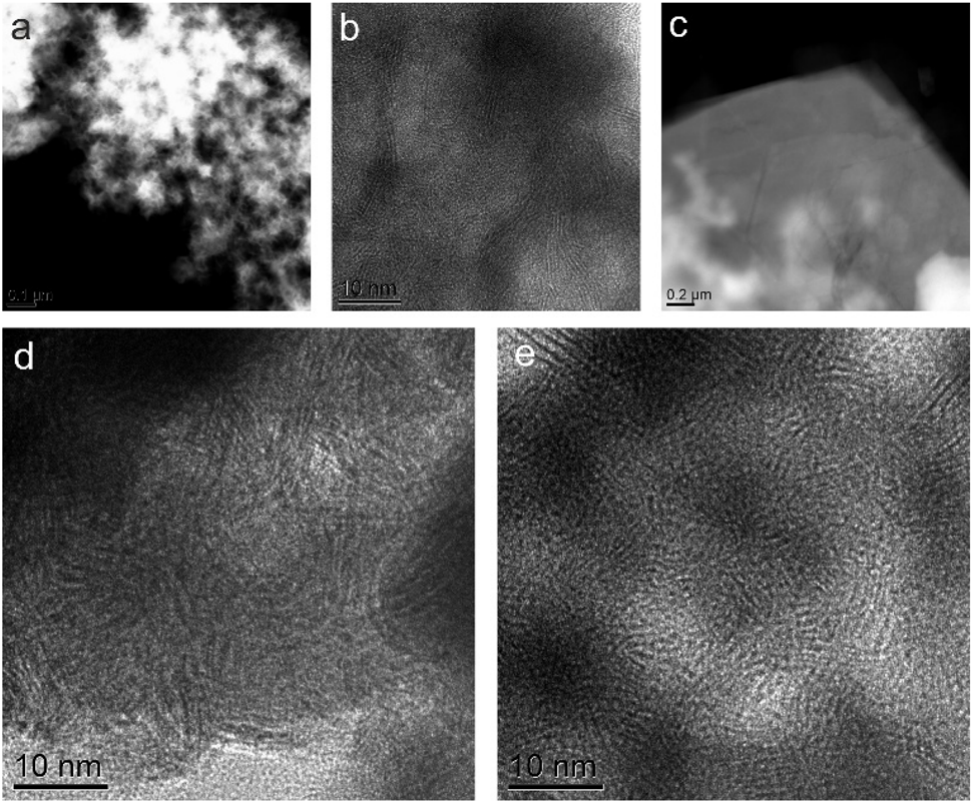
TEM analysis of WSe_2_, Mo_2_Ti_2_C_2_ MXene, and the Mo_2_TiC_2_/WSe_2_ hybrid. (a) HAADF-STEM image and (b) HRTEM image of WSe_2_ nanoflowers. (c) HAADF STEM image of a Mo_2_Ti_2_C_2_ MXene flake. (d and e) HRTEM images of the Mo_2_TiC_2_/WSe_2_ hybrid.

Scanning Electron Microscopy (SEM) and Transmission Electron Microscopy (TEM) analyses were employed to determine the morphology and elemental composition of the materials (Fig. 2). The SEM image of pristine WSe_2_ (Fig. 2A) exhibits its characteristic flower-like aggregates, which is typical of the bottom-up preparation approach. This is also confirmed by HAADF image (Fig. 3a). HRTEM image (Fig. 3b) shows nano size layered crystal features embedded in amorphous structure. In contrast, the MXene displays its typical layered architecture (Fig. 2B and 3c). The corresponding images of the hybrid confirm the coexistence of both components (Fig. 2c). Furthermore, EDS mapping verifies the distribution of the constituent elements throughout the hybrid structure, confirming the successful preparation of the heterostructure (Fig. 2C). HRTEM images (Fig. 3d and e) of the hybrid material show similar nano size layered crystal features embedded in amorphous structure.

**Fig. 4:**
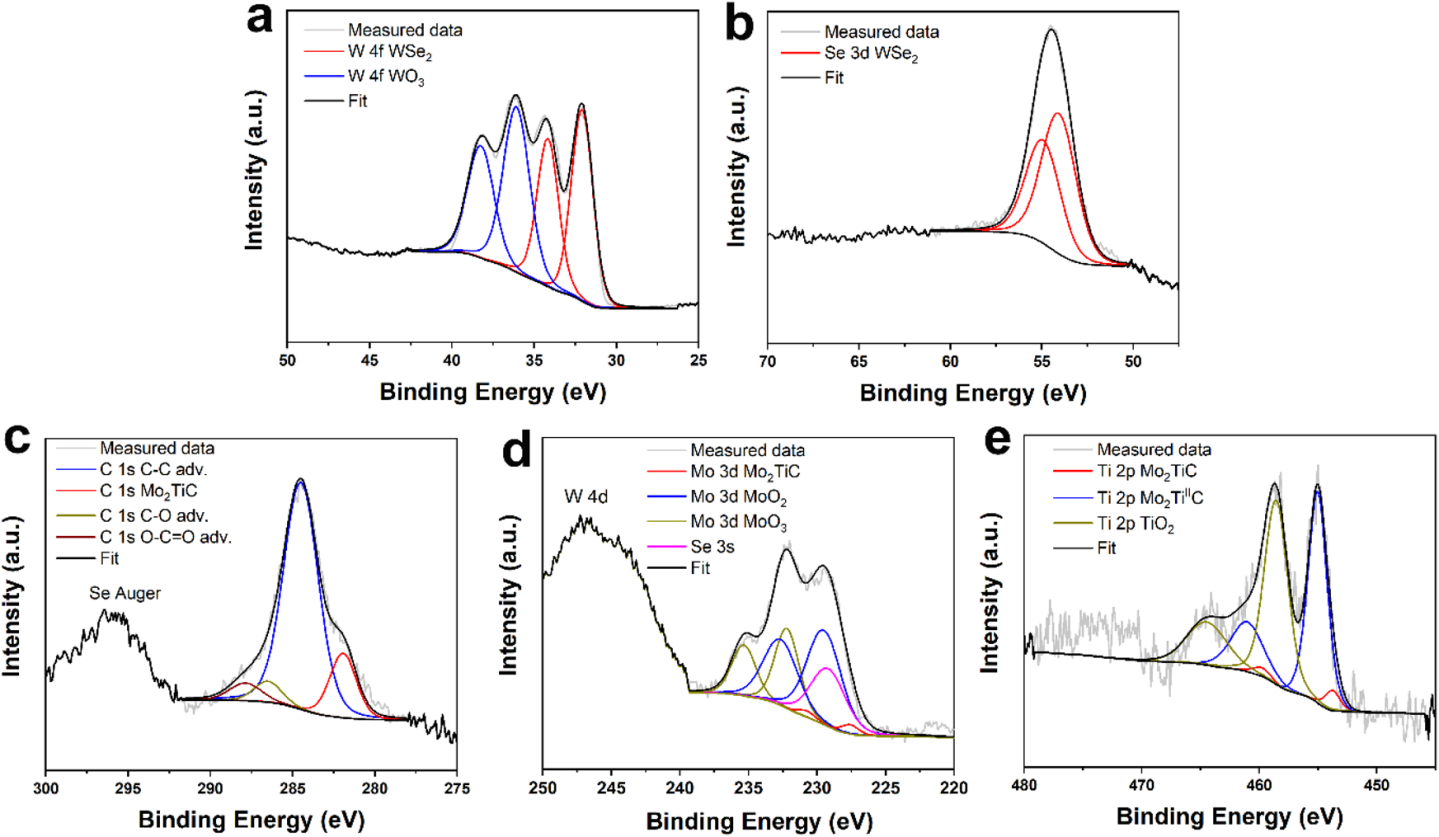
(a) Deconvoluted X-ray photoelectron spectra of Mo_2_TiC_2_/WSe_2_ showing (a) W 4f and (b) Se 3d, (c) C 1s, (d) Mo 3d and (e) Ti 2p chemical states.

Surface composition analysis was performed by X-ray photoelectron spectroscopy (XPS). The XPS survey spectra of the Mo_2_TiC_2_/WSe_2_ hybrid and the reference materials (WSe_2_ and Mo_2_TiC_2_) are shown in Fig. S2. High-resolution (deconvoluted) XPS spectra for WSe_2_ are shown in Fig. S3. The high-resolution spectrum of Se 3d consisted of two peaks associated with Se^II^ (53.7 and 54.6 eV for Se 3d_5/2_ and 3d_3/2_, respectively). The W 4f high-resolution spectrum was comprised of two doublets assigned to WSe_2_ (31.6 and 33.7 eV for W 4f_7/2_ and W 4f_5/2_, respectively) and WO_3_ (35.5 and 37.7 eV for W 4f_7/2_ and W 4f_5/2_, respectively). The presence of WO_3_ clearly indicates surface oxidation of the material, a phenomenon commonly observed in tungsten-based TMDs and widely reported in the literature. Such surface oxide species have been shown to enhance the electrocatalytic performance of the material.^25,26,37,38^ Mo_2_TiC was analyzed in an identical manner, with XPS data shown in Fig. S4. For Mo 3d high-resolution spectrum, an extensive surface oxidation was observed, which is documented by the presence of two doublets originating from MoO_2_ (229.6 and 233.0 eV for Mo 3d_5/2_ and Mo 3d_3/2_, respectively) and MoO_3_ (232.5 and 235.7 eV for Mo 3d_5/2_ and Mo 3d_3/2_, respectively). A doublet associated with the Mo_2_TiC MXene phase was also identified in the Mo 3d spectrum (227.5 and 230.8 eV for Mo 3d_5/2_ and Mo 3d_3/2_, respectively). These results show a high degree of surface oxidation, however, Mo oxides, particularly MoO_2_ have been previously associated with good catalytic performance for HER.^39,40^ For the Ti 2p spectrum, three doublets associated with Ti in MXene (454 and 460 eV for Ti 2p_3/2_ and Ti 2p_1/2_, respectively), slightly oxidized Ti in MXene (455.8 and 462.0 eV for Ti 2p_3/2_ and Ti 2p_1/2_, respectively) and TiO_2_ (459.5 and 465.5 eV for Ti 2p_3/2_ and Ti 2p_1/2_, respectively), were identified as previously reported in other reports.^41^ Finally, in the C 1s spectrum, a carbide peak at 282.5 eV together with peaks originating from adventitious contamination, were also identified. The XPS spectra of the hybrid material are shown in Fig. 4. A comparison of the high-resolution spectra from this material to the pure parent materials revealed no significant changes in terms of peak positions. Slight changes in the ratios between individual peaks were identified (see Tables S3–S6), but these likely originate from a small degree of variance in the surface composition of the materials. Such results indicate a synergistic effect between both materials, rather than dramatic compositional changes introduced by the synthesis of the hybrid.

**Fig. 5:**
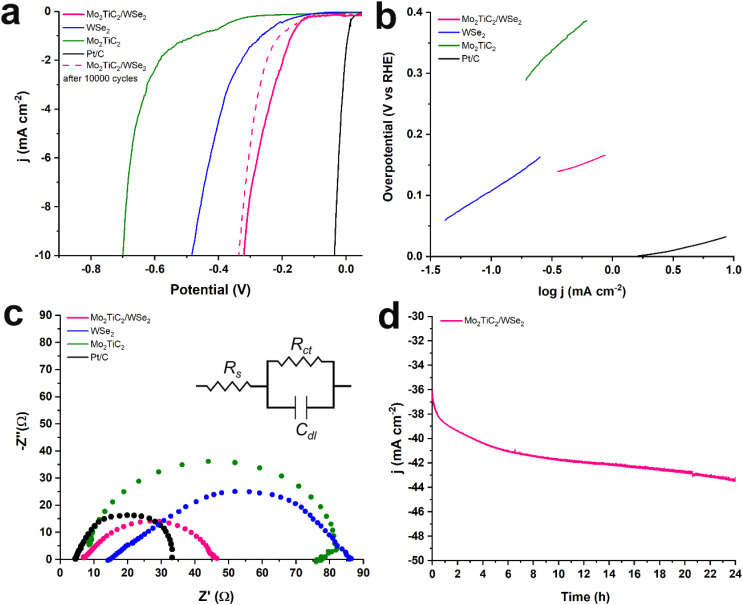
(a) iR-corrected LSVs for HER obtained at 1600 rpm rotation speed and 5 mV s^−1^ scan rate before (solid lines) and after 10 000 cycles (dashed lines) in Ar-saturated aqueous 0.5 M H_2_SO_4_, (b) Tafel slopes, and (c) Nyquist plots for Mo_2_TiC_2_/WSe_2_ (pink), Mo_2_TiC_2_ MXene (green), WSe_2_ (blue), and Pt/C (black). Data were fitted using a Randles equivalent circuit. (d) Chronoamperometric response of Mo_2_TiC_2_/WSe_2_ at −1.56 V (*vs.* RHE) for 24 h.

Next, the HER performance of Mo_2_TiC_2_/WSe_2_ hybrid electrocatalyst was evaluated, alongside reference materials WSe_2_, Mo_2_TiC_2_ MXene, and 20 wt% Pt/C, using LSV measurements in an Ar-saturated 0.5 M H_2_SO_4_ aqueous electrolyte (Fig. 5). The Mo_2_TiC_2_/WSe_2_ hybrid shows markedly superior HER activity (Fig. 5a), initiating hydrogen evolution at −0.14 V *vs.* RHE, which is 120 and 410 mV lower than WSe_2_ and Mo_2_TiC_2_ MXene, respectively. At −10 mA cm^−2^, it delivers a low overpotential of 320 mV, outperforming WSe_2_ and Mo_2_TiC_2_ MXene by 160 mV and 380 mV, as the pristine components exhibit substantially higher overpotentials of −0.48 V and −0.70 V *vs.* RHE.

The enhanced reaction kinetics of Mo_2_TiC_2_/WSe_2_, corroborated by Tafel and EIS data, can be attributed to the direct contact enabled by the robust deposition of WSe_2_ onto Mo_2_TiC_2_ MXene, facilitating efficient electron transfer within the hybrid structure. Additionally, the abundant flower-like WSe_2_ in the hybrid provides a high surface area with more exposed active edge sites for enhanced HER performance.

In order to obtain information about the reaction mechanism, we extracted Tafel slopes from LSV curves (Fig. 5b) and performed electrochemical impedance spectroscopy (EIS) (Fig. 5c). The Mo_2_TiC_2_/WSe_2_ hybrid exhibited the lowest Tafel slope, 74 mV dec^−1^, showing that the Heyrovsky step is rate-limiting. Protons first adsorb on the surface (Volmer step) and then combine to form H_2_. In contrast, pristine WSe_2_ and Mo_2_TiC_2_ MXene had higher slopes, 128 and 191 mV dec^−1^, pointing to slower kinetics.

EIS at −2 mA cm^−2^ further corroborate the above findings by revealing a markedly reduced charge-transfer resistance for the Mo_2_TiC_2_/WSe_2_ hybrid. The hybrid's charge-transfer resistance was 55 Ω, while reference materials displayed higher resistance values of 70 Ω for WSe_2_ and 75 Ω for Mo_2_TiC_2_. This improvement derives from: (i) the direct contact between WSe_2_ and Mo_2_TiC_2_, which helps electron transfer, and (ii) the flower-like WSe_2_, which exposes more active sites for HER. The electrochemically active surface area (ECSA) was calculated from the double-layer capacitance (*C*_dl_) from CV curves measured in the non-faradaic range (50–500 mV s^−1^, Fig. S5). In reference capacitance, 40 µF cm^−2^ was used to estimate ECSA values, which were 16.5 cm^2^ for Mo_2_TiC_2_/WSe_2_, 1.8 cm^2^ for Mo_2_TiC_2_, 3.9 cm^2^ for WSe_2_. The larger surface area of the hybrid reflects a higher number of accessible active sites. Interestingly, Mo_2_TiC_2_/WSe_2_ exhibited an ECSA value of 10.8 cm^2^ after 10 000 cycles, showing a slight decrease from the original value. Additionally, the specific activity was calculated as *j*_ECSA_ = (*j*_geo_ × *A*_geo_)/ECSA where *j*_geo_ is the geometric current density and *A*_geo_ the geometric surface area of the electrode. The Mo_2_TiC_2_/WSe_2_hybrid shows a lower *j*_ECSA_ than the pristine materials, indicating that the improvement in HER performance mainly arises from its increased electrochemically active surface area and the high density of exposed active sites rather than intrinsically higher catalytic activity. Table S1 summarizes the electrocatalytic parameters for all tested materials. As summarized in Table S2, the HER performance of the Mo_2_TiC_2_/WSe_2_ hybrid is comparable to other WSe_2_- and MXene-based electrocatalysts reported in the literature, supporting its potential as an efficient and stable system.

Furthermore, the stability of the hybrid was evaluated by running 10 000 cycles, as shown in Fig. 5a. After continuous cycling, the hybrid exhibited only a minor potential increase of about 20 mV. To further examine its durability, chronoamperometric measurements were conducted at −1.56 V *vs.* RHE, corresponding to a current density of approximately −36 mA cm^−2^, for 24 h under 1600 rpm (Fig. 5d). The measurement showed an initial activation process, during which the current density progressively increased in magnitude, reaching approximately −43 mA cm^−2^ after 24 h of continuous operation. This gradual increase reflects surface activation and demonstrates the excellent long-term durability of the Mo_2_TiC_2_/WSe_2_ hybrid under prolonged electrolysis conditions. Post-HER SEM imaging reveals that the hybrid retains its overall morphology, with slightly more exposed Mo_2_TiC_2_ sheets resulting from the partial detachment of loosely bound WSe_2_ nanoflowers during gas evolution (Fig. S6). The observed increase in oxygen is attributed mainly to residual Nafion and surface rehydration. No oxide-related structural changes are detected, confirming the stability of both components under HER conditions. Similar behavior is observed in the post-chronoamperometry SEM analysis (Fig. S7), further confirming the structural robustness of the Mo_2_TiC_2_/WSe_2_ hybrid under prolonged electrochemical operation.

## Conclusions

In summary, we developed Mo_2_TiC_2_/WSe_2_ nanoarchitectures through a simple solvothermal route, achieving intimate interfacial contact between the Mo_2_TiC_2_ MXene and WSe_2_ nanoflowers. The hybrid exhibited a low overpotential (−0.32 V at −10 mA cm^−2^), a Tafel slope of 74 mV dec^−1^ consistent with a Volmer–Heyrovsky pathway, and a significantly enlarged electrochemically active surface area. The electrocatalyst exhibited excellent stability, with negligible performance loss after 10 000 cycles and a gradual activation during 24 h chronoamperometry at −36 mA cm^−2^, reaching ∼−43 mA cm^−2^. These results highlight Mo_2_TiC_2_/WSe_2_ as an efficient and durable electrocatalyst for hydrogen evolution.

## Conflicts of interest

There are no conflicts to declare.

## Supplementary Material

NA-OLF-D5NA01182E-s001

## Data Availability

Data analysed during the study are accessible *via* the Zenodo repository https://zenodo.org/records/17157686. Supplementary information (SI): imaging, spectroscopic and electrocatalytic data. See DOI: https://doi.org/10.1039/d5na01182e.
