# Supplementary material for: Mo2TiC2/WSe2 nanoarchitectures: in situ grown nanoflowers for efficient hydrogen electrocatalysis
Source: Nanoscale Adv. 2026 Feb 11;8(6):1844–50. doi: 10.1039/d5na01182e (PMC12939973; doi:10.1039/d5na01182e)
Supplement: NA-008-D5NA01182E-s001 [file NA-008-D5NA01182E-s001.pdf]

Supplementary Information (SI) for Nanoscale Advances.
This journal is © The Royal Society of Chemistry 2026

Supporting Information

Mo_2TiC_2/WSe_2 Nanoarchitectures: In-situ Grown Nanoflowers for Efficient Hydrogen Electrocatalysis

Antonia Kagkoura,[a] Sergii A. Sergiienko,[a,b] Anastasios Papavasileiou,[a] Jan Luxa,[a] Zhongquan Liao[c] and Zdeněk Sofer*[a]*

[a]*Department of Inorganic Chemistry, University of Chemistry and Technology Prague, Technická 5, 166 28 Prague 6, Czech Republic*

[b]*Centre for Advanced Materials Application, Slovak Academy of Sciences, Dúbravská cesta 5807/9, 84511, Bratislava, Slovakia*

[c]*Department of Microelectronic Materials and Nanoanalysis, Fraunhofer Institute for Ceramic Technologies and Systems IKTS, Maria-Reiche-Str. 2, 01109 Dresden, Germany*

*Email: kagkourn@vscht.cz, soferz@vscht.cz

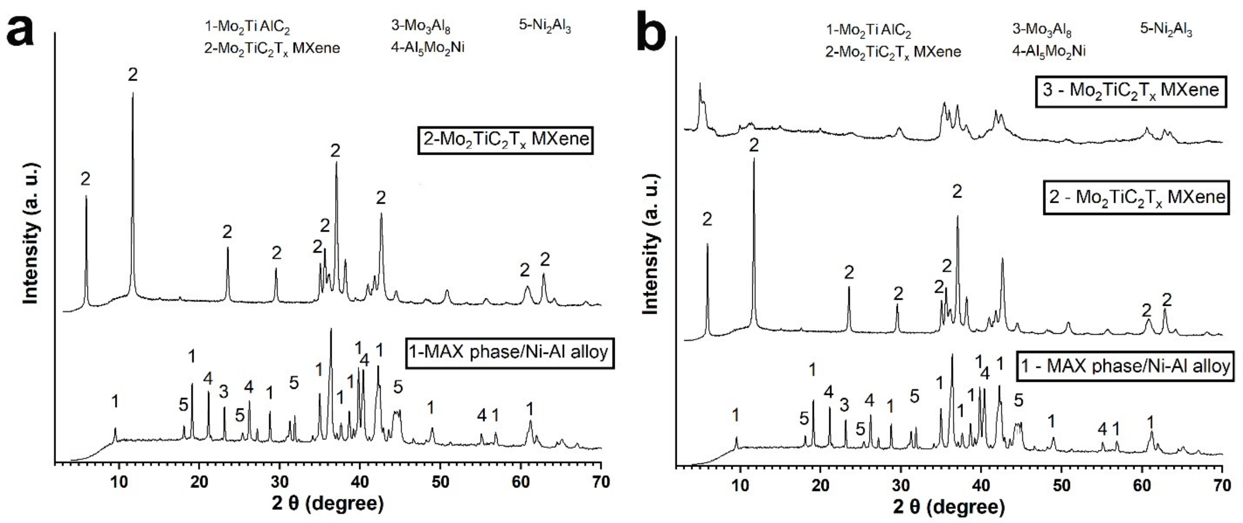

Figure S1. XRD patterns for (a) Mo_2TiAlC_2 MAX phase (1) and (b) $Mo_2TiC_2T_x$ MXene after etching (2) and after delamination (3).

.

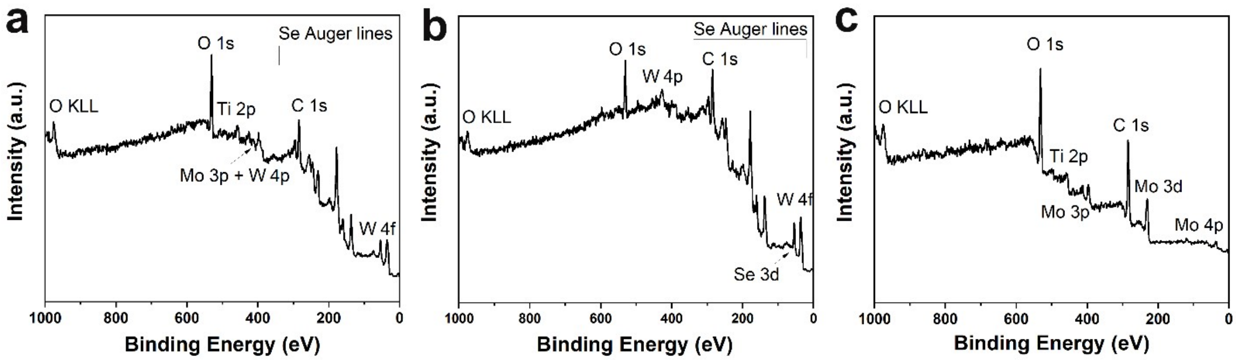

Figure S2. XPS survey spectra of (a) Mo_2TiC_2/WSe_2, (b) WSe_2 and (c) Mo_2TiC_2 MXene.

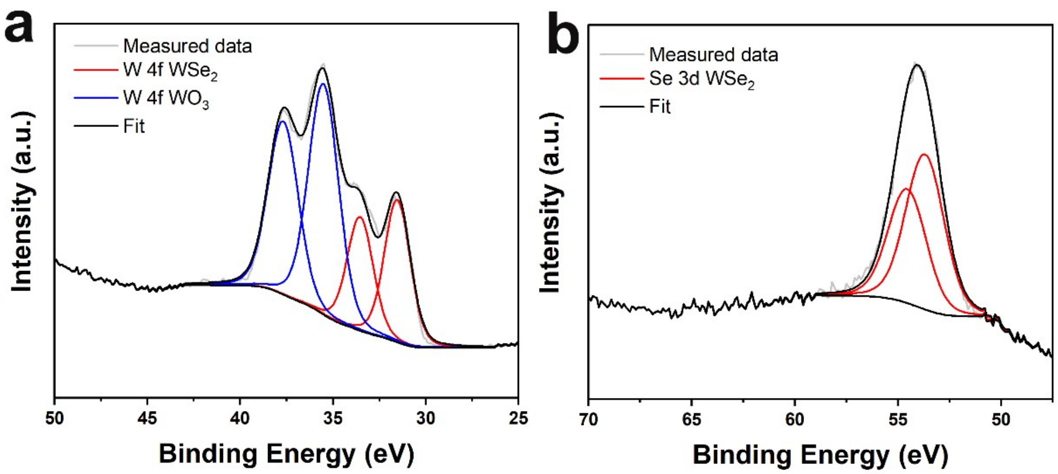

Figure S3. Deconvoluted X-ray photoelectron spectra of WSe_2 displaying (a) W 4f and (b) Se 3d chemical states.

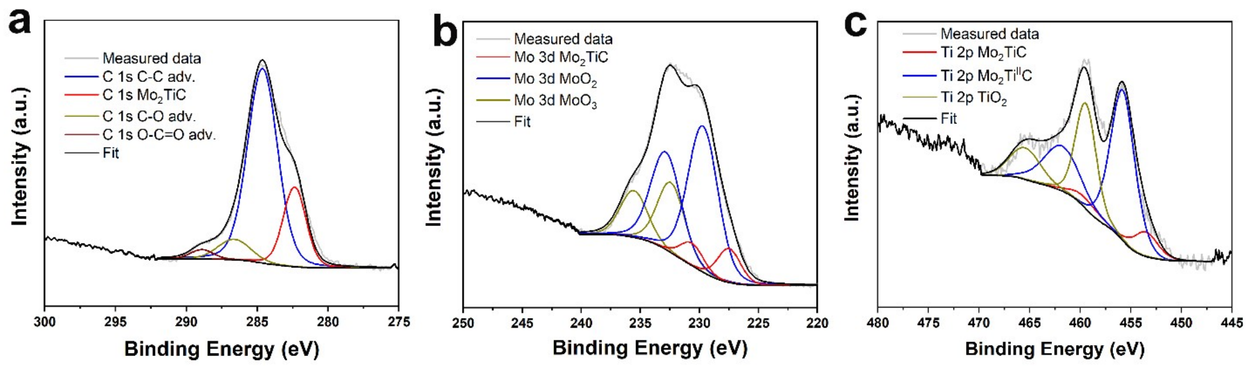

Figure S4. Deconvoluted X-ray photoelectron spectra of Mo_2TiC_2 MXene displaying (a) C 1s, (b) Mo 3d and (c) Ti 2p chemical states.

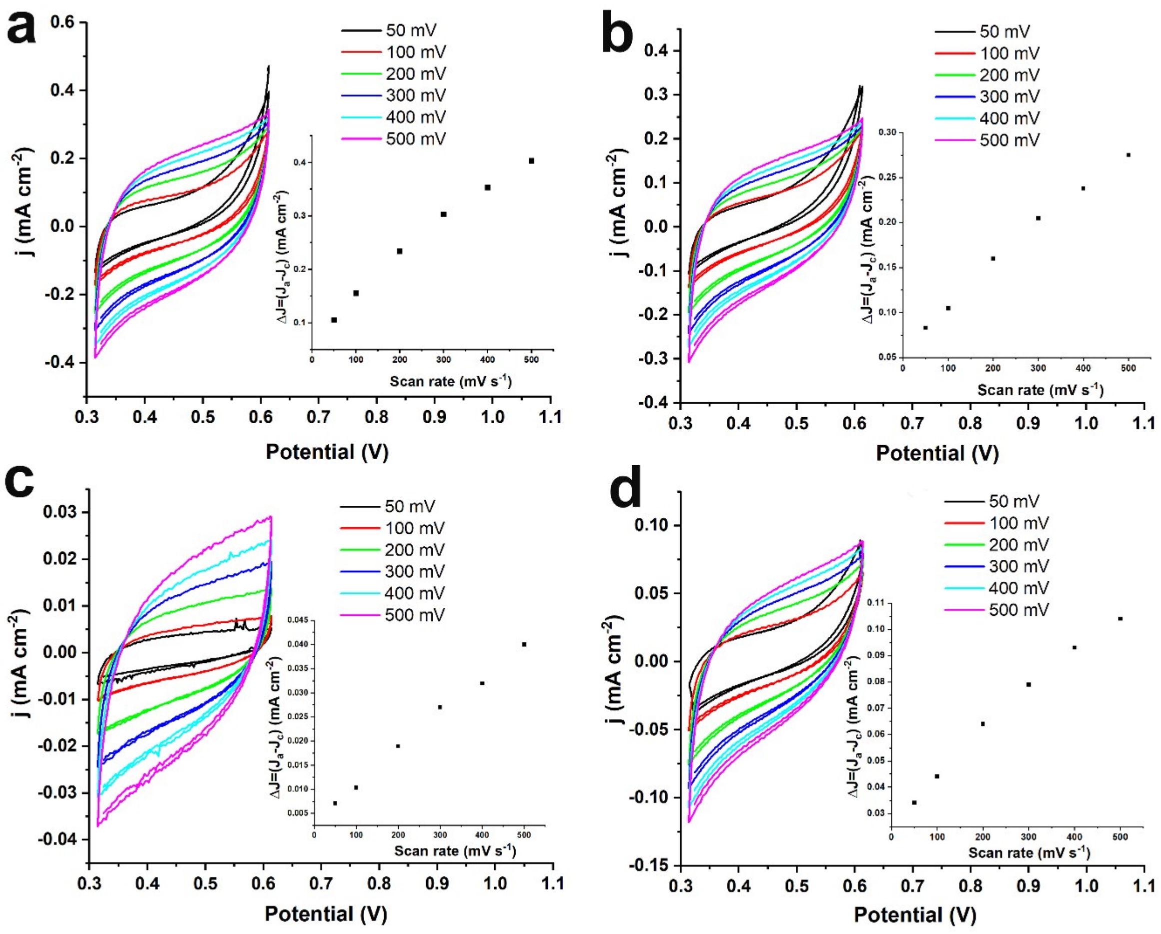

Figure S5. Cyclic voltammograms of (a) Mo_2TiC_2/WSe_2, (b) Mo_2TiC_2/WSe_2 after 10000 cycles, (c) Mo_2TiC_2 MXene and (d) WSe_2 in an argon-saturated aqueous 0.5 M H_2SO_4 electrolyte, at a rotation speed of 1600 rpm and scan rates from 50 to 500 mV s^{-1}. Inset: Scan rate dependence of the current densities for the corresponding materials.

Table S1. HER parameters for all tested materials.

Material	Onset potential (V vs RHE)	Potential (V vs RHE) at -10 mA cm^{-2}	Tafel slope (mV dec^{-1})	Rct (Ω)	ECSA (cm^2)	j_{ECSA} (mA/cm^2 $_{ECSA}$)
Mo_2TiC_2/WSe_2	-0.14	-0.32	74	55	16.5	-0.0038
Mo_2TiC_2/WSe_2[a)]	-0.15	-0.33	77	-	10.8	-
WSe_2	-0.26	-0.48	128	65	3.9	-0.0241
Mo_2TiC_2	-0.55	-0.7	191	70	1.8	-0.0762
Pt/C	-0.019	-0.037	31	29	-	-

a) after 10000 cycles

Table S2. Comparison Table of WSe_2 and MXene-based electrocatalysts.

Material	Electrolyte	Potential (V vs RHE) at -10 mA cm^{-2}	Tafel slope (mV dec^{-1})	R_{ct} (Ω)	Ref.
Mo_2TiC_2/WSe_2	0.5 M H_2SO_4	-0.32	74	55	This work
3% Ni–WSe_2	0.5 M H_2SO_4	-0.28	85	850	1
1T′-WSe_2	0.5 M H_2SO_4	-0.47	104	294	2
WS_2/WSe_2	0.5 M H_2SO_4	-0.291	57	6.37	3
$WSe_2/Ti_3C_2Cl_2$	0.5 M H_2SO_4	-0.19	50	18.6	4
MoS_2/Ti_3C_2	0.5 M H_2SO_4	-0.280	68	14.74	5
Ti_3C_2 NWs	0.5 M H_2SO_4	-0.476	129	7.08	6
$Mo_2TiC_2T_x$	0.5 M H_2SO_4	-0.35	112	32.75	7
WSe_2-CoP	0.5 M H_2SO_4	-0.33	133	75	8
Co–WSe_2@PPy	1 M KOH	-0.337	138	307	9
Co-WSe_2@PANI	1 M KOH	-0.308	127	139	9
Co-Ti_3C_2	1 M NaOH	-0.30	147	-	10
Cl-$Ti_3C_2Cl_2$	1 M KOH	-0.259	92	0.313	11
HF-$Ti_3C_2Cl_2$	1 M KOH	-0.444	311	0.44	11

Table S3. Concentration of individual states deconvoluted from high-resolution C 1s spectra.

Sample	C 1s at.% (Mo_2TiC)	C 1s at.% (C-C adv.)	C 1s at.% (C-O adv.)	C 1s at.% (O=C-O adv.)
Mo_2TiC_2	21.3	68.2	7.9	2.6
Mo_2TiC_2/WSe_2	15.9	73.4	5.4	5.3

Table S4. Concentration of individual states deconvoluted from high-resolution W 4f spectra.

Sample	W 4f at.% (WSe_2)	W 4f at.% (WO_3)
WSe_2	35.3	64.7
Mo_2TiC_2/WSe_2	42.3	57.7

Table S5. Concentration of individual states deconvoluted from high-resolution Mo 3d spectra.

Sample	Mo 3d at.% (Mo_2TiC_x)	Mo 3d at.% (MoO_2)	Mo 3d at.% (MoO_3)	Se 3s (at. %)
Mo_2TiC_2	11.1	61.9	27.0	-
Mo_2TiC_2/WSe_2	2.5	50.2	28.8	18.5

Table S6. Concentration of individual states deconvoluted from high-resolution Ti 2p spectra.

Sample	Ti 2p at.% (Mo_2TiC)	Ti 2p at.% ($Mo_2Ti^{II}C$)	Ti 2p at.% (TiO_2)
Mo_2TiC_2	9.4	56.1	34.5
Mo_2TiC_2/WSe_2	3.7	45.7	50.6

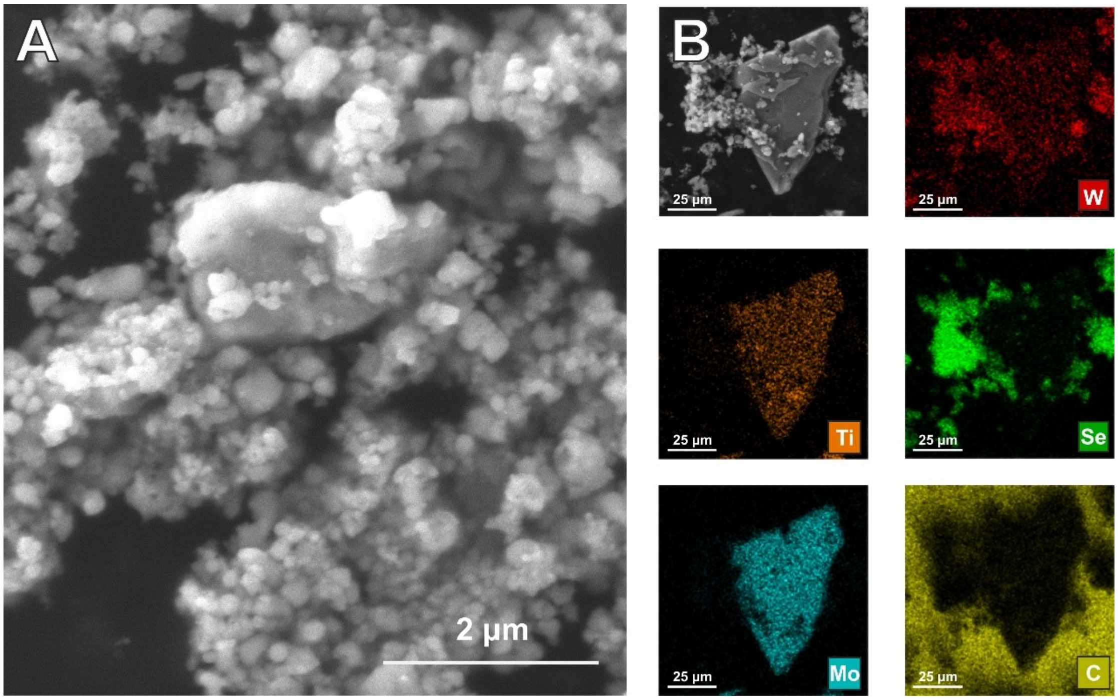

Figure S6. SEM image of (A) Mo_2TiC_2/WSe_2 hybrid and (B) Mo_2TiC_2/WSe_2 hybrid with corresponding EDS elemental maps (a-c) showing the distribution of elements.

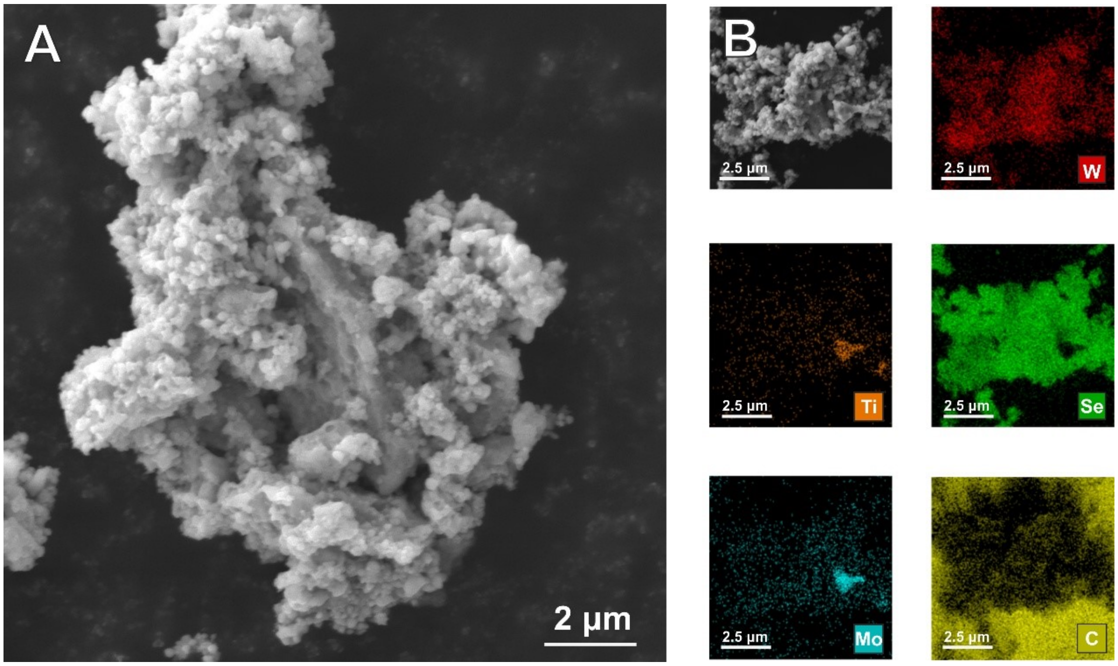

Figure S7. SEM image of (A) Mo_2TiC_2/WSe_2 hybrid after chronoamperometry and (B) Mo_2TiC_2/WSe_2 hybrid after chronoamperometry with corresponding EDS elemental maps showing the distribution of elements.

References

1 S. R. Kadam, A. N. Enyashin, L. Houben, R. Bar-Ziv and M. Bar-Sadan, *J. Mater. Chem. A*, 2020, **8**, 1403–1416.

2 A. Debnath, N. Sen, A. Das, S. Bhattacharjee, S. Dey, B. Satpati and K. K. Chattopadhyay, *Appl. Phys. Lett.*, 2024, **125**, 091903.

3 B. Rehman, K. M. M. D. K. Kimbulapitiya, M. Date, C.-T. Chen, R.-H. Cyu, Y.-R. Peng, M. Chaudhary, F.-C. Chuang and Y.-L. Chueh, *ACS Appl. Mater. Interfaces*, 2024, **16**, 32490–32502.

4 A. Kagkoura, A. Papavasileiou, S. Wei, Filipa. M. Oliveira, J. Šturala and Z. Sofer, *NPJ 2D Mater. Appl.*, 2025, **9**, 73.

5 L. Huang, L. Ai, M. Wang, J. Jiang and S. Wang, *Int. J. Hydrogen Energy*, 2019, **44**, 965–976.

6 W. Zhao, B. Jin, L. Wang, C. Ding, M. Jiang, T. Chen, S. Bi, S. Liu and Q. Zhao, *Chinese Chem. Lett.*, 2022, **33**, 557–561.

7 J. Luxa, P. Kupka, F. Lipilin, J. Šturala, A. Subramani, P. Lazar and Z. Sofer, *ACS Catal.*, 2024, **14**, 15336–15347.

8 A. Kagkoura, C. Stangel, R. Arenal and N. Tagmatarchis, *Nanomaterials*, 2023, **13**, 35.

9 S. Cogal, G. Celik Cogal, M. Mičušík, M. Kotlár and M. Omastová, *Int. J. Hydrogen Energy*, 2024, **49**, 689–700.

10 J. Wang, Y. Liu and G. Yang, *Mater. Res. Express*, 2019, **6**, 025056.

11 B. Sarfraz, M. T. Mehran, M. M. Baig, S. R. Naqvi, A. H. Khoja and F. Shahzad, *Int. J. Energy Res.*, 2022, **46**, 10942–10954.
